# Grading of invasive breast carcinoma through Grassmannian VLAD encoding

**DOI:** 10.1371/journal.pone.0185110

**Published:** 2017-09-21

**Authors:** Kosmas Dimitropoulos, Panagiotis Barmpoutis, Christina Zioga, Athanasios Kamas, Kalliopi Patsiaoura, Nikos Grammalidis

**Affiliations:** 1 Information Technologies Institute, Centre for Research and Technology Hellas, Thessaloniki, Greece; 2 Department of Pathology, Agios Pavlos General Hospital of Thessaloniki, Greece; University of South Alabama Mitchell Cancer Institute, UNITED STATES

## Abstract

In this paper we address the problem of automated grading of invasive breast carcinoma through the encoding of histological images as VLAD (Vector of Locally Aggregated Descriptors) representations on the Grassmann manifold. The proposed method considers each image as a set of multidimensional spatially-evolving signals that can be efficiently modeled through a higher-order linear dynamical systems analysis. Subsequently, each H&E (Hematoxylin and Eosin) stained breast cancer histological image is represented as a cloud of points on the Grassmann manifold, while a vector representation approach is applied aiming to aggregate the Grassmannian points based on a locality criterion on the manifold. To evaluate the efficiency of the proposed methodology, two datasets with different characteristics were used. More specifically, we created a new medium-sized dataset consisting of 300 annotated images (collected from 21 patients) of grades 1, 2 and 3, while we also provide experimental results using a large dataset, namely BreaKHis, containing 7,909 breast cancer histological images, collected from 82 patients, of both benign and malignant cases. Experimental results have shown that the proposed method outperforms a number of state of the art approaches providing average classification rates of 95.8% and 91.38% with our dataset and the BreaKHis dataset, respectively.

## Introduction

**Fig 1:**
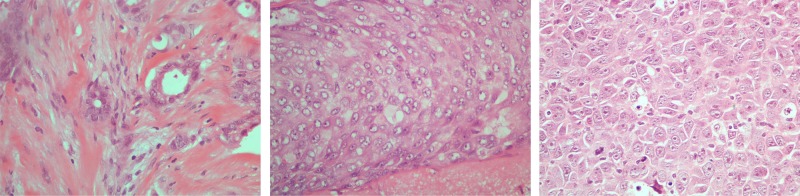
Indicative cases of H&E breast cancer histological images of (a) Grade 1, (b) Grade 2 and (c) Grade 3.

Breast cancer is the second most common cancer in the world and by far the most frequent cancer among women [1, 2]. According to the American Cancer Society, it is estimated that, only in 2017, there will be approximately 255,180 new cases of invasive breast cancer and around 41,070 deaths from breast cancer (for both women and men) in the U.S. [3]. Detection and diagnosis of breast cancer can be achieved by mammography or ultrasound for the identification of suspicious regions of the breast, followed by a tissue biopsy and microscopic examination for the determination of the presence and grade of cancer. During the visual examination of the biopsy specimen of the tissue, pathologists look for certain features that can help them predict disease prognosis, i.e., how likely the cancer is to grow and spread. These features include the spatial arrangement of the cells, morphological characteristics of the nuclei (nuclear pleomorphism), whether they form tubules (tubule formation) and how many of the neoplastic cells are in the process of dividing (mitotic index). These histologic features taken together determine the extent or spread of cancer at the time of the diagnosis and are known as “Nottingham Grading System”. The grading of the invasive breast carcinoma is classified into a three-point scale: Grade 1 (low grade, well-differentiated carcinoma), Grade 2 (intermediate grade, moderately differentiated carcinoma) and Grade 3 (high grade, poorly-differentiated carcinoma) [4], as shown in Fig 1.

However, the visual qualitative assessment is a labor and time-consuming task [5] and results in inter- and intra-observer variation in diagnosis, i.e., different pathologists may come up with diverse interpretations, leading to different diagnoses, or the same pathologist may make different diagnosis at different times for the same set of histological images [6]. In other words, the main problem in histological grading of breast carcinoma is not only the identification of the correct combination of features and the morphological heterogeneity within the tumor, but also the inter-observer variations in the assessment of the subjective criteria[5, 7]. The recent advances, however, on whole-slide scanning systems have enabled the digitization of glass slides with stained tissue sections at high resolutions and have offered new opportunities to image processing techniques to quantify histopathologic procedures and support pathologists in the interpretation of histological images. To this end, various methods [8] of automatic Breast Cancer (BC) grading have been proposed in the literature in order to increase the accuracy and reproducibility of diagnosis. In most of the cases the main challenges are the accurate segmentation [9] and detection of histologic primitives, such as nuclei, as well as the extraction of a number of suitable textural or spatial features in order to model the pathologist’s knowledge used in clinical practice. On the other hand other approaches have used deep-learning techniques [10] aiming to address the problem by extracting knowledge directly from the data. However, the training of complex deep learning networks requires a large number of images, i.e., large datasets, as well as considerable effort and time for their annotation by expert pathologists.

In this paper, we propose a novel approach for the grading of invasive breast carcinoma, which considers each histological image as a set of multidimensional spatially-evolving signals that can be efficiently represented as a cloud of points in a non-Euclidean space, such as a Grassmann manifold. In contrast to traditional approaches that attempt to model pathologist’s knowledge, the proposed method aims to model directly the available data, i.e., histological images, avoiding the detection of histologic primitives through a series of preprocessing steps. Experimental results show that the proposed method provides high detection rates both with small and large datasets, outperforming a number of state of the art approaches. More specifically, the contributions of this paper are summarized as follows: i) We introduce a new methodology for the modelling of static breast cancer histological images through higher-order linear dynamical systems analysis. ii) We demonstrate that each histological image can be represented as a cloud of points on the Grassmann manifold and we propose the VLAD encoding of each image on the non-Euclidean space. iii) To evaluate the efficiency of the proposed methodology, we created a new dataset of 300 annotated images of grades 1–3 [11], while we also provide experimental results using the well-known BreaKHis dataset [12, 13] containing 7,909 breast histological images of both benign and malignant cases.

The remaining of this paper is organized as follows: In Section 2, similar works on breast cancer histological image analysis are presented, while in Section 3, we describe the material used in the experimental analysis, as well as the proposed methodology for the automated grading of invasive breast carcinoma. Finally, the experimental results of our study are given in Section 4, while conclusions are drawn in Section 5.

## Related work

Numerous methods have been proposed in the literature for the detection of breast cancer in histological images[8, 14–18]. As was mentioned above, most of them focus mainly on the segmentation and identification of histologic primitives, such as nuclei, and the extraction of suitable features. Doley *et al*. [19] introduced a methodology for the automated grading of breast cancer histological images using spectral clustering with textural (Gabor, Grey Level and Haralick) and architectural (Voronoi diagram, Delaunay triangulation, minimum spanning tree, nuclear characteristics) features, yielding an accuracy of 93.3% in a dataset of 48 breast biopsy tissue studies. On the other hand, Niwas et al. [20] extracted color textural features for breast cancer diagnosis using log-Gabor wavelet transform and least square support vector machine (LS-SVM) classifier. More recently, Kowai et al. [21] studied different clustering algorithms for the segmentation of nuclei and extracted various morphological, topological and textural features for the classification of 500 microscopic images in two classes, benign or malignant, while Filipczuk *et al*. [22] applied a circular Hough transform for the identification of nuclei and then extracted a set of features for the classification of biopsies by using four different classifiers. Similarly, George *et al*. [23] applied a nuclei segmentation approach and then studied several neural network architectures to investigate the most suitable network model for classifying the tumor effectively. On the other hand, Zhang *et al*. [24] presented a classification scheme based on a one-class kernel principle component analysis model ensemble using various features extracted from a gray level co-occurrence matrix. Finally, Spanhol *et al*. [12] created a big dataset of 7,909 breast cancer histopathology images, including both benign and malignant images (but without grading annotation), and tested different texture descriptors and state of the art classifiers.

Nevertheless, the majority of the above methods is feature dependent and in many cases involves a series of pre-processing steps, such as segmentation, nuclei separation and detection, which affect significantly the final classification result. The need for methods that will be able to learn directly from data has led many researchers to apply more sophisticated techniques, such as deep learning networks. More specifically, Cruz-Roa *et al*. [14] proposed a method for the automatic detection of invasive ductal carcinoma in whole slide images using Convolutional Neural Networks (CNN), while Spanhol *et al*. [10] presented a method based on the extraction of image patches for training the CNN and the combination of these patches for the final classification of images into two classes, benign or malignant. Similarly, Litjens *et al*. [25] investigated the general applicability of CNNs to improve the efficiency of cancer diagnosis in H&E images by applying it to two tasks: the detection of prostate cancer in biopsy specimens and the detection of breast cancer metastases in resected sentinel lymph nodes. The aforementioned methods showed high classification rates, however, the training of the complex deep learning networks requires a large number of images for the accurate determination of their parameters. To this end, in this paper, we propose a non-customized method for the grading of invasive breast carcinoma, i.e., the proposed method is also applicable to other contexts, which provides promising results for both small and large datasets. The proposed method is not based on the detection of histologic primitives, as is usually done in traditional methods, but it models directly the breast cancer histological images as a set of spatially-evolving multidimensional signals, which are mapped and encoded on the Grassmann manifold.

## Material and methods

### Dataset description

The proposed methodology was tested and evaluated on two datasets with different characteristics. The first one is a medium-sized dataset containing breast cancer histological images of different grades, while the second one is a large dataset that includes both benign and malignant (without grading information) images. More specifically, the first dataset [11] was created for the scope of this paper and contains archival cases of breast carcinoma histological specimens received at the Department of Pathology, “Agios Pavlos” General Hospital of Thessaloniki, Greece. As was also confirmed by the Scientific Council of the hospital, there was no need for ethical approval for this study, since all samples were analyzed anonymously and were collected during the routine course of care for diagnostic purposes. In the typical hospital workflow, breast tumor excisions or biopsies are performed in the operating room and, then, the material is sent for processing to the Pathology Department. The samples were fixed in buffered formalin and then embedded in paraffin. From the paraffin blocks, sections with a thickness of 4 μ m were cut using a microtome and mounted on glass slides. In order to be able to visualize the structures of interest in the tissue, the sections were dyed with Hematoxylin and Eosin (H&E) stain, as routine stain according to bioethics rules, and the glass slides were coverslipped. Then, the glass slides were visually examined by a Pathologist using routine light microscopy. The scoring was given by one Pathologist and reviewed by another one using the Scarff-Bloom-Richardson histological grading system, Nottingham modification. Score of 3 (Grade 3) was given by the Pathologist to images showing marked variation of histologic features upon comparison with normal tissue, a score of 2 (Grade 2) for moderate variations and a score of 1 (Grade 1) for mild variations (score was given by the Pathologist firstly to the slides and secondly to images). Our dataset consists of 300 images (Grade 1: 107, Grade 2: 102 and Grade 3: 91 images) of resolution 1280x960 corresponding to 21 different patients with invasive ductal carcinoma of the breast. The image frames were from regions afflicted by tumor growth captured through a Nikon digital camera attached to a compound microscope with x40 magnification objective lens.

**Fig 2:**
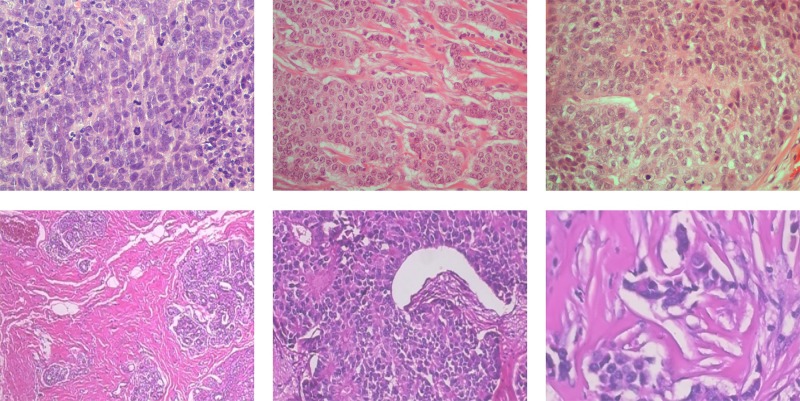
(a)-(c) Indicative cases of H&E breast cancer histological images from our dataset (image resolution 1280x960) and (d)-(f) malignant cases from the BreaKHis dataset (image resolution 700x460) with different magnification factors:(d) x40, (e) x100 and (f) x200.

The second dataset is the publicly available BreaKHis database [12, 13] consisting of 7,909 breast cancer histological images acquired on 82 patients. The dataset contains microscopic biopsy images of benign and malignant breast tumors with no grading information, i.e., it contains two different classes of images. The samples were generated from breast tissue biopsy slides stained with hematoxylin and eosin, while images (with resolution 700x460) were acquired using magnifying factors of x40 (625 benign and 1370 malignant), x100 (644 benign and 1437 malignant), x200 (623 benign and 1390 malignant) and x400 (588 benign and 1232 malignant). In Fig 2, we indicatively present malignant cases from both our dataset and the BreaKHis dataset.

### Methodology

**Fig 3:**
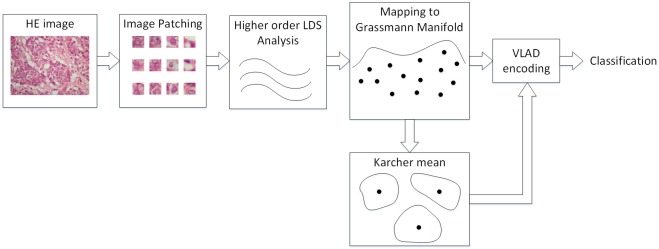
The proposed methodology.

Breast cancer histological images contain spatially evolving characteristics and interrelated patterns, which are strongly related to the grading of invasive breast carcinoma. For this reason, the proposed method attempts to model the histological images as a set of multidimensional spatially-evolving signals that can be efficiently represented as a cloud of Grassmannian points, enclosing the dynamics and appearance information of the image. By taking advantage of the geometric properties of the space in which these points lie, i.e., the Grassmann manifold, we estimate the VLAD encoding [26] of each image on the manifold in order to identify the grading of invasive breast carcinoma, as shown in Fig 3.

#### The dynamical model

Towards this end, we initially attempt to model histological images through a linear dynamical system analysis. Linear dynamical systems have been widely used in the past for the modeling and analysis of time-series in a broad range of applications in engineering (e.g., dynamic texture analysis or human action recognition[27–29]), as well as economics and social sciences. A linear dynamical system (LDS) is associated with a first order ARMA process with white zero mean IID Gaussian input and for this reason LDSs are also known as linear Gaussian state-space models. In general, LDS models attempt to associate the output of the system, i.e., the observation, with a linear function of a state variable, while in each time instant, the state variable depends linearly on the state of the previous time instant. Both state and output noise are zero-mean normally distributed random variables and apart from the output of the system, all other variables (state and noise variables) are hidden. More specifically, the stochastic modeling of the signal’s dynamics and appearance is encoded by two stochastic processes, in which dynamics are represented as a time-evolving hidden state process *x*(*t*) ∈ *R*^*n*^ and the observed data *I*(*t*) ∈ *R*^*d*^ as a linear function of the state vector:

x(t+1)=Ax(t)+Bv(t) (1)

$$I(t)=\bar{I}+Cx(t)+w(t)$$ (2)

where *A* ∈ *R*^*nxn*^ is the transition matrix of the hidden state and *C* ∈ *R*^*dxn*^ is the mapping matrix of the hidden state to the output of the system. The quantities *w*(*t*) and *Bv*(*t*) are the measurement and process noise respectively, with *w*(*t*)*~N(0*,*R)* and *Bv*(*t*) *~N(0*,*Q)*, while $\bar{I}$ is the mean value of observations.

To apply such a time-series analysis approach to a static breast cancer histological image, we initially divide each image into a number of image patches, since describing each image by local descriptors is preferable to a holistic representation. Then, we consider each patch as a multidimensional signal evolving in the spatial domain, i.e., in consecutive pixels *i*, instead of discrete time instances *t*. For the estimation of the system parameters, i.e., *A* and *C*, several approaches have been proposed based either on Expectation-Maximization (EM) algorithm or non-iterative subspace methods [30]. Since these approaches require high computational cost, a suboptimal method was proposed in [31], according to which the columns of the mapping matrix *C* can be considered as an orthonormal basis, e.g., a set of principal components. However, most of the approaches in the literature often make a simplifying assumption of the data structure, which leads to the concatenation of data into a simple vector. In order to fully exploit any hidden correlation between the different channels of data, i.e., the RGB data of a histological image, we use a third order tensor representation for each *NxN* patch, i.e., *Y* ∈ *R*^*NxNx*3^. Subsequently, we apply a generalization of the singular value decomposition for higher order tensors, such as higher-order SVD analysis as proposed in[32–33].

$$Y=S\times_{1}U_{\left(1\right)}\times_{2}U_{\left(2\right)}\times_{3}U_{\left(3\right)}$$ (3)

where *S* ∈ *R*^*N*×*N*×3^ is the core tensor, while *U*_(1)_ ∈ *R*^*N*×*N*^, *U*_(2)_ ∈ *R*^*N*×*N*^ and *U*_(3)_ ∈ *R*^3×3^ are orthogonal matrices containing the orthonormal vectors spanning the column space of the matrix and ×_j_ denotes the *j-*mode product between a tensor and a matrix. Since the columns of the mapping matrix *C* of the stochastic process need to be orthonormal, we can easily choose one of the three orthogonal matrices of Eq (3) to be equal to *C*. In addition, given the fact that the choice of matrices *A*, *C* and *Q* in Eqs (1) and (2) is not unique, we can consider *C = U*_*(3)*_ and

$$X=S\times_{1}U_{\left(1\right)}\times_{2}U_{\left(2\right)}$$ (4)

Hence, Eq (3) can be reformulated as follows:

$$Y=X\times_{3}C\Leftrightarrow Y_{\left(3\right)}=CX_{\left(3\right)}$$ (5)

where Y_(3)_ and X_(3)_ indicate the unfolding along the third dimension of tensors *Y* and *X* respectively, and *X*_(3)_ = [*x*(1),*x*(2),…,*x*(*n*)] are the estimated states of the system. If we define *X*_1_ = [*x*(2),*x*(3),…,*x*(*n*)] and *X*_2_ = [*x*(1),*x*(2),…,*x*(*n*−1)] the transition matrix *A*, containing the dynamics of the signal, can be easily computed by using least squares as:

$$A=X_{2}X_{1}^{T}(X_{1}X_{1}^{T}{)}^{-1}$$ (6)

**Fig 4:**
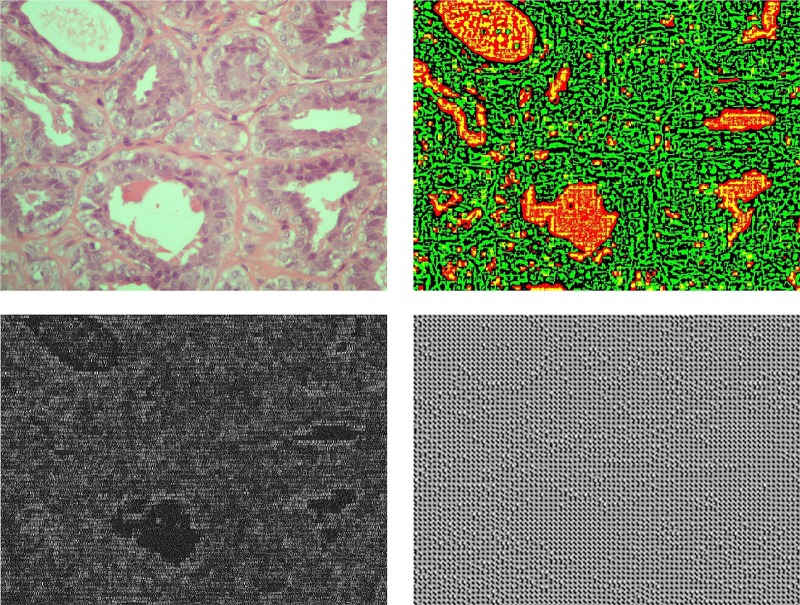
Visualization of the higher-order linear dynamical system on an H&E stained breast cancer histological image using patches of size 16x16. (a) Input image, (b) hidden state, (c) transition matrix *A* and (d) mapping matrix *C*.

Fig 4 illustrates the observation data, i.e., the original breast cancer histological image considering image patches of size 16x16 (for visualization purposes, we have used non-overlapping patches), as well as the corresponding hidden state variables and the transition and mapping matrices *A* and *C*, respectively, for each image patch.

In order to ensure the stability of the system, the spectral radius of the transition matrix *A* needs to be smaller than 1, i.e., |*λ*_1_(*A*)| ≤ 1, where *λ*_1_ denotes the first eigenvalue of matrix *A*, considering the eigenvalues in descending order of magnitude. Towards this end, we apply an approximation solution based on a convex optimization technique [34], which leads to the estimation of the stabilized transition matrix *A* through the solving of the following quadratic problem:

$$\begin{array}{l} minimize\;\alpha P\alpha -2q^{T}\alpha +r\\ subject\;to\;g^{T}\alpha \leq 1 \end{array}$$ (7)

where *α* = *vec*(*A*), $q=vec(X_{1}X_{2}^{T})$, $r=tr(X_{2}^{T}X_{2})$ and $P=I⊗(X_{1}^{T}X_{1})$. Here, *I* is the identity matrix, *tr*(∙) indicates the trace of a matrix, *vec*(∙) operator converts a matrix to vector and ⊗ denotes the Kronecker product. In order to estimate the stabilized transition matrix, i.e., *α* = *vec*(*A*), we define $g=vec(u_{1}v_{1}^{T})$ with vectors *u*_1_ and $v_{1}^{T}$ corresponding to the first eigenvalue of the transition matrix *A*, i.e., *A* = *UΣV*^*T*^ or $\lambda_{1}=u_{1}^{T}Av_{1}$.

**Fig 5:**
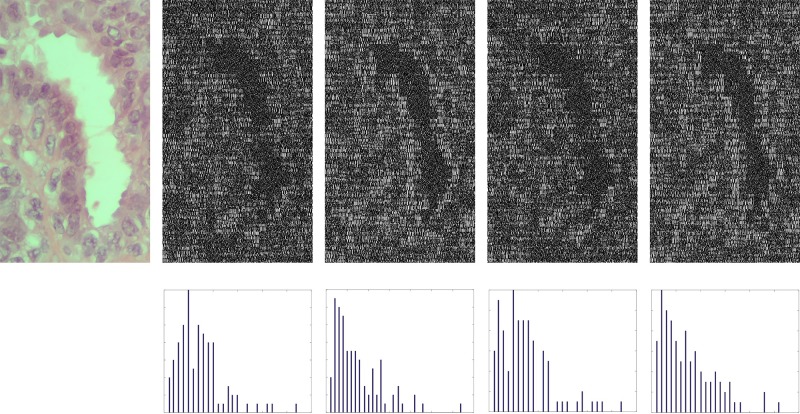
(a) An indicative portion of a histological image consisting of 32x16 patches. Each image patch is modeled by four higher-order linear dynamical systems corresponding to the four possible directions of the signal’s evolution. Figures (b)-(e) illustrate the stabilized transition matrices of each patch in the four directions, i.e., (b) right, (c) up, (d) left, and (e) down. Figures (f)-(i) illustrate the corresponding histograms of the stabilized higher–order LDS descriptors.

For the selection of patches in a breast cancer histological image, different strategies can be adopted, e.g., overlapping patches, non-overlapping patches or random selection of patches (in Section 4, experimental results with different patching strategies and various sizes are presented in detail). In order to fully exploit image patches information, we consider the spatial evolution of multidimensional signals towards all possible directions, i.e., right, left, up and down. To do so, we rotate each image patch by ninety degrees in clockwise direction for three consecutive times, so that each patch is finally modeled by four higher order linear dynamical systems corresponding to the four possible directions of the signal’s evolution. Fig 5 illustrates the estimated stabilized transition matrices *A* containing the dynamics information towards the four possible directions of signal’s transmission and the corresponding histograms of the stabilized higher–order LDS descriptors.

#### Grassmannian analysis

Having modeled each image patch using a higher-order linear dynamical systems approach, our next step is to represent the parameters of each dynamical system, *M = (A*,*C)*, as a point on the space of the extracted descriptors. Towards this end, we initially estimate the finite observability matrix of each dynamical system, $O_{m}^{T}(M)=[C^{T},{\left(CA\right)}^{T},{\left(CA^{2}\right)}^{T},\ldots ,{\left(CA^{m-1}\right)}^{T}]$ (in our experiments, we set *m* equal to 3), and, then, we apply a Gram-Scmidt orthonormalization [35] procedure in order to represent each descriptor with an orthogonal matrix *G* ∈ *R*^*mNx*3^. The columns of this matrix contain an orthonormal basis and for this reason we can consider that *G* corresponds to a point on the Grassmann manifold, i.e., a quotient of the special orthogonal group SO(n). Since it can be shown that SO(n) is a Riemannian manifold [36], we can claim that a Grassmann manifold, i.e., a manifold with linear subspaces, is endowed with a Riemannian structure.

For the modeling of a breast cancer histological image, we apply a vector representation approach, which aggregates the descriptors, i.e., the extracted Grassmannian points, based on a locality criterion on the manifold. More specifically, in this paper we adopt the Vector of Locally Aggregated Descriptors (VLAD) encoding approach and we attempt to apply it to the space created by the parameters of the stabilized higher-order linear dynamical systems. VLAD representation is considered as a simplified coding scheme of the earlier Fisher Vector (FV) representation and has shown to outperform histogram representations in bag of features approaches [37]. In general, VLAD encoding considers only the first-order differences and assigns descriptors to a single mixture component. More specifically, let us consider a codebook, ${\left\{m_{i}\right\}}_{i=1}^{k}=\{m_{1},m_{2},\ldots ,m_{k}\}$, with *k* visual words and local descriptors *x*, where each descriptor is associated to its nearest codeword. The VLAD descriptor, $\bar{V}$, is created by concatenating the *k* local difference vectors ${\left\{u_{i}\right\}}_{i=1}^{k}$ corresponding to differences *m*_*i*_−*x*_*j*_, with *m*_*i*_ = *NN*(*x*_*j*_), where *x*_*j*_ are the descriptors belonging to class *i*, with *i* = 1,…,*k*. In other words, the VLAD vector of an image can be estimated as follows:

$$\bar{V}={\left\{u_{i}\right\}}_{i=1}^{k}=\{u_{1},\ldots ,u_{k}\}=\left\{\sum_{\begin{array}{c} x_{j}\;such\;that\\ m_{1}=NN(x_{j}) \end{array}}\left(m_{1}-x_{j}\right),\ldots ,\sum_{\begin{array}{c} x_{j}\;such\;that\\ m_{k}=NN(x_{j}) \end{array}}\left(m_{k}-x_{j}\right)\right\}$$ (8)

while the final VLAD code is determined by the *L*_*2*_-normalization of vector $\bar{V}$:

$$\bar{V}=\bar{V}/{\left\|\bar{V}\right\|}_{2}$$ (9)

However, the main problem of applying such an approach to our case lies in the fact that the descriptors extracted from the image patches, i.e., the Grassmannian points, do not lie in the Euclidean space. Hence, in order to represent breast cancer histological images through a VLAD encoding approach, we need first to resolve two significant problems. First, we should define a dissimilarity metric between two descriptors on the manifold, in order to estimate the difference between a codeword and a Grassmannian point, and, second, we have to define a suitable notion of the "mean" between a finite set of points on the manifold.

To address the first problem, we take advantage of the Riemannian structure of our manifold in order to define the distance between two points as the Riemannian distance between two subspaces, i.e., the distance corresponding to the length of the shortest geodesic connecting two Grassmannian points. To do so, we apply the inverse exponential map between two points on the manifold, e.g., *G*_1_ and *G*_2_, to map the first Grassmannian point on a tangent space of the second one, while preserving the distance between the points. In other words, using the inverse exponential map, we can move from a Grassmann manifold to an Euclidean space, such as the tangent space of a manifold's point. Hence, the dissimilarity metric between *G*_1_ and *G*_2_, can be defined as follows:

$$d(G_{1},G_{2})={\left\|{exp}_{G_{2}}^{-1}G_{1}\right\|}_{F}$$ (10)

where the inverse exponential map, *exp*^−1^, defines a vector in the tangent space of a manifold's point, i.e., the mapping of *G*_1_ to the tangent space of *G*_2_, and ||.||_F_ indicates the Frobenius norm. For more details regarding the estimation of inverse exponential map on Grassmann manifold, we refer the reader to [38].

On the other hand, for the definition of the *k* representative words, i.e., the *k* means in Eq (8), of the codebook ${\left\{m_{i}\right\}}_{i=1}^{k}$, we initially identify the most representative *k* Grassmannian points among the existing points on the manifold, by using a *K*-Medoid approach, and then we use the estimated medoids for the initialization of the Karcher mean algorithm [39]. This procedure enables us to ensure the deterministic convergence of the algorithm, since we avoid picking points at random, as is commonly done for the initialization of the Karcher mean algorithm. Based on the estimated Karcher means, *m*_*i*_, we can re-identify the members *x*_*j*_ of each class, i.e., *m*_*i*_ = *NN*(*x*_*j*_), using the dissimilarity metric defined in Eq (10). Hence, the VLAD encoding of a histological image on the Grassmann manifold for a codebook of *k* representative words, ${\left\{m_{i}\right\}}_{i=1}^{k}$, can be defined as:

$$\bar{V}=\frac{1}{\left\|\bar{V}\right\|}\left\{\sum_{\begin{array}{c} G_{j}\;such\;that\\ the\;Karcer\;mean\\ m_{1}=NN(G_{j}) \end{array}}{\left\|{exp}_{m_{1}}^{-1}G_{j}\right\|}_{F},\ldots ,\sum_{\begin{array}{c} G_{j}\;such\;that\\ the\;Karcer\;mean\\ m_{k}=NN(G_{j}) \end{array}}{\left\|{exp}_{m_{k}}^{-1}G_{j}\right\|}_{F}\right\}$$ (11)

For the classification of a breast cancer histological image the VLAD representation on Grassmann manifold is estimated and the extracted code is provided to an SVM classifier to infer the grading of invasive breast carcinoma.

## Results and discussion

This section provides the details about the experiments conducted for the evaluation of the proposed method. The goal of this experimental evaluation is twofold: i) we aim to validate the efficiency of the proposed methodology in the grading of invasive breast carcinoma using a medium-sized dataset that contains breast cancer histological images of three different grades, i.e., grades 1, 2 and 3, and ii) we attempt to demonstrate the reproducibility of our method using a large dataset containing both benign and malignant images (without including any grading information), acquired with four different magnification factors (x40, x100, x200 and x400). Experimental results involve comparison of the proposed method with 14 different state of the art methods, including deep learning and handcrafted feature based methods (relying on textural, graph or morphological features).

### Grading of invasive breast carcinoma

**Fig 6:**
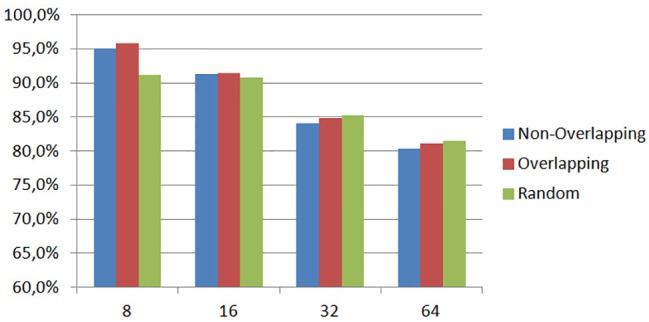
Classification rates with different patch sizes and patching strategies.

In this section, we evaluate the performance of the proposed method in the grading of invasive breast carcinoma using our dataset [11], which contains breast cancer histological images of three different grades. For the classification results presented in this section, we estimated the classification rate as the ratio between the correctly classified images, *N*_*c*_, and the total number of images, *N*_*all*_, in the dataset:

To define the best parameters of our method, we initially carried out experiments with different patch sizes and patching strategies, i.e., overlapping, non-overlapping or random patches. For all experiments we adopted a 5-fold cross validation approach and we estimated the average classification rate of the five trials using Eq (12). As we can clearly see in Fig 6, patches of small size, i.e., 8x8, provide the best classification rates (95.8% for overlapping, 95.1% for non-overlapping and 91.2% for random patches) with overlapping patches obtaining the higher detection rate. Results show that patches of 8x8 size contain sufficient dynamics and appearance information for the classification of histological images with resolution 1280x960, while at the same time, the strategy of overlapping patches (with 50% overlap between patches) results in an adequate number of Grassmannian points i.e., 151,376 points corresponding to all possible multidimensional spatial signals in each histological image.

**Fig 7:**
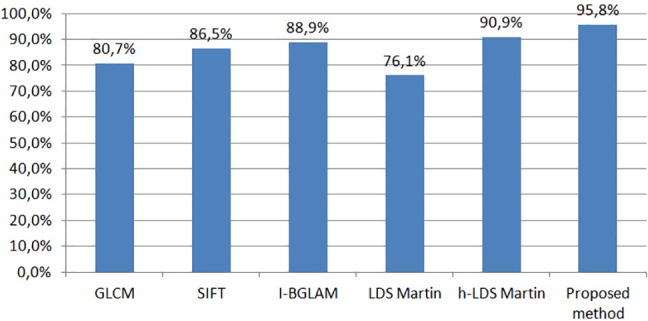
Comparison of the proposed method against five state of the art approaches based on textural features.

After the definition of the optimal parameters of our method, in the next experiment we aim to show that the use of a third order tensor for the representation of image patches along with the VLAD encoding on the Grassmann manifold improve the classification accuracy of the standard LDS [31] descriptor. More specifically, for the standard LDS descriptor we adopted a bag-of-features approach based on subspace angles (in this case the Martin distance [40] was used as a similarity metric between two LDS descriptors as in [41]) and then, we followed the same bag of features approach using the higher-order LDS descriptor. Experimental results in Fig 7 show that the proposed method achieves an improvement of 19.07% compared to the standard LDS descriptor, while the use of VLAD encoding, instead of Martin distance, improves the classification accuracy up to 4.9% (h-LDS Martin: 90.9%, proposed method: 95.8%). In addition, in Fig 7 we compare the proposed method with three other state of the art approaches, such as standard GLCM, i-BGLAM [42] and SIFT [43], that have been used in the past in various image classification problems. The experimental results show again that the proposed method, due to its ability to model the hidden dynamics of spatial signals in the image patches, outperforms all other approaches achieving improvements of 15.1%, 9.3% and 6.9% with respect to GLCM, SIFT and i-BGLAM, respectively.

**Fig 8:**
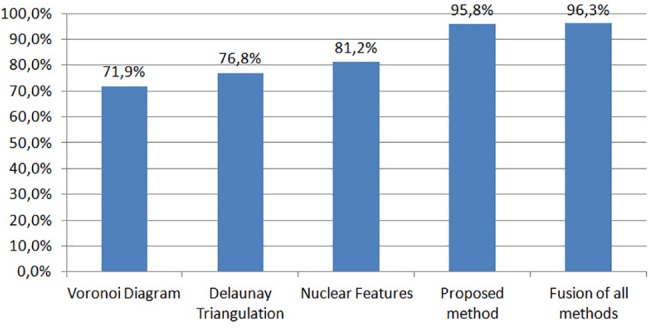
Comparison of the proposed method against two graph-based approaches, i.e., Voronoi diagram and Delaunay triangulation and a method based on various nuclear features. The last classification rate corresponds to the fusion of the proposed method with the three other approaches.

Finally, we evaluated the performance of the proposed method against three other approaches, which are based on the extraction of graph features and nuclear features after a preprocessing step for the detection of nuclei in each histological image. More specifically, the first two approaches rely on the extraction of two different sets of graph features aiming to model the arrangement of nuclei within a histological image. The first set of features is based on Voronoi Diagram [44], while the second one on Delaunay Triangulation, as proposed in [18]. On the other hand, the third method is based on the extraction of a number of shape and textural features of nuclei, e.g. nuclear density, nuclear shape regularity, number and size of nucleoli, as proposed in our previous work [45]. For the experimental results of the methods in Fig 8, we have used as a preprocessing step for the detection of nuclei (the preprocessing step includes segmentation of nuclei and splitting of clustered nuclei) the methodology proposed in our previous work [6], while for the classification of the extracted features we used the same SVM classifier with radial basis function kernel for all methods to infer the label of classes. As we can see in Fig 8, the proposed method outperforms all methods based on nuclei characteristics. We can also notice that by fusing the proposed method with graph (both Voronoi Diagram and Delaunay Triangulation) and nuclear features, the classification accuracy increases slightly by 0.5%.

### Classification of benign and malignant cases

**Fig 9:**
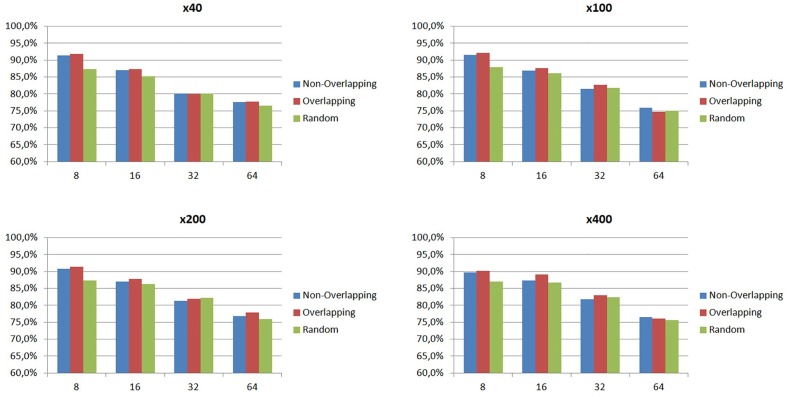
Classification rates with different patch sizes and patching strategies for the four magnifications factors. (a) x40, (b) x100, (c) x200, (d) x400.

In this section, we aim to evaluate the efficiency of the proposed method using a large dataset, such as BreaKHis, consisting of 7,909 breast cancer histological images (with resolution 700x460) of two classes: benign and malignant. To define the optimum size of patches for each magnification factor, we run experiments with four different patch sizes, 8, 16, 32 and 64, and three different patching strategies, as in the previous section. For the results in Fig 9, we divided the BreaKHis dataset into training (70%) and testing (30%) sets, as proposed in [10], and we estimated the average of five trials using Eq (12) (the patients used to build the training set are not used for the testing set). The same procedure was applied independently to each of the four magnifications available in the dataset.

Fig 9 displays the classification performance of our method for each magnification factor, using different patch sizes and patching strategies. As we can see, the best experimental results are produced again using overlapping patches (50% overlap) with a small size, i.e., 8x8, independently of the magnification factor. More specifically, the highest average classification rates, 91.8% and 92.1%, are produced with magnification factors of x40 and x100, respectively, while the other two magnification factors (x200 and x400) provide also classification rates higher than 90%, i.e., 91.4% and 90.2% respectively. We have to note here that the optimal classification rate could be achieved by selecting all possible patches from each image, however, this would increase the computation cost. We believe that the use of overlapping patches, with 50% of overlap between patches, is a reasonable compromise, since it results in the extraction of an adequate number of patches, i.e., 9634, 2270, 552 and 110 for patch sizes of 8x8, 16x16, 32x32 and 64x64 respectively. For the random patches, in our experiments we fixed the arbitrary number of patches to 10,000 for patch size of 8x8, 2,500 for patch size of 16x16 and 1,000 for the other two patch sizes. However, one could also run experiments with more random patches in order to further increase the classification rate.

To compare the performance of our methodology against the state of the art algorithms presented in [10] and [12], we adopted the same experimental protocol followed in these works to ensure a fair comparison. More specifically, we estimated the patient score as:

$$Patient\;Score=\frac{N_{c}}{N_{p}}$$ (12)

where N_c_ is the number of correctly classified images for each patient and N_P_ are the number of cancer images of patient *P*. Similarly, the global patient classification rate is defined as follows [10]:

$$Global\;Patient\;Classification\;Rate=\frac{\sum Patient\;Score}{Total\;number\;of\;Patients}$$ (13)

**Fig 10:**
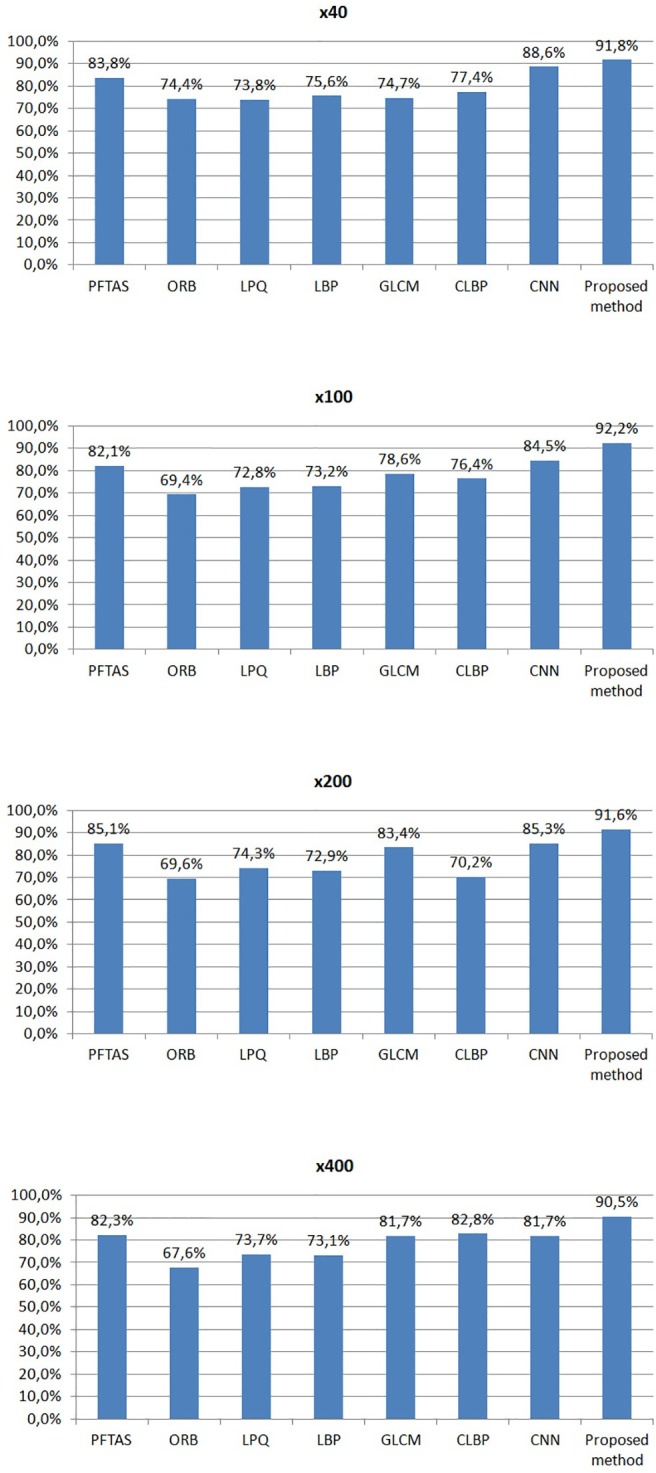
The patient classification rates of the proposed method and seven state of the art methods using histopathological images of different magnification factors. (a) x40, (b) x100, (c) x200 and (d) x400.

For the experimental results in Fig 10, we estimated the average global patient classification rate of five trials, as proposed in [10] and [12]. The first six classification approaches, i.e., Local Binary Patterns (LBP) [46], Completed Local Binary Patterns (CLBP) [47], Local Phase Quantization (LPQ) [48], Gray Level Co-Occurrence Matrices (GLCM) [49], Parameter-Free Threshold Adjacency Statistics (PFTAS) [50] and Oriented FAST and Rotated BRIEF (ORB) [51], are based on different textural descriptors, while the last one, i.e., Convolutional Neural Networks (CNN) [10], is a deep learning approach. As we can see from Fig 10, the proposed method outperforms all state of the art approaches yielding average patient classification rates of 91.8%, 92.2%, 91.6% and 90.5% for magnification factors of x40, x100, x200 and x400, respectively. We have to note here that the classification rates presented in Fig 10 are the best classification rates for each method, i.e., the rates corresponding to the optimal set of parameters for each method.

**Fig 11:**
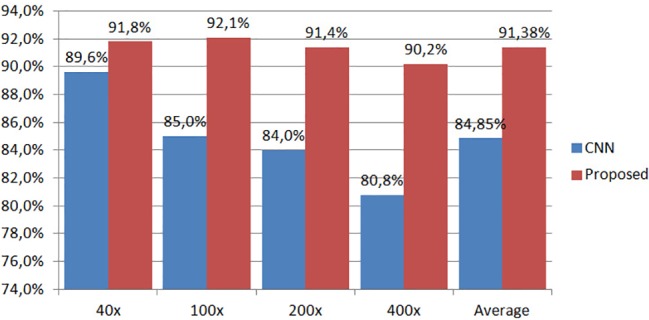
The average image classification rates of the proposed method and the CNN-based deep learning approach presented in [10].

Finally, in Fig 11 we present a comparative analysis of the proposed method against the second most efficient approach, i.e., the CNN-based deep learning approach [10], using as a metric the image classification rate defined in (12). We can see again that the proposed method outperforms the CNN-based approach in all magnification factors showing its great potential even with a large dataset. More specifically, the proposed method achieves improvements up to 2.2%, 7.1%, 7.4% and 9.4% for magnification factors of x40, x100, x200 and x400, respectively, i.e., improvement of 6.53% in the average classification rate (proposed method: 91.38%, CNN: 84.85%). For the experimental results of our method using both datasets, we did not apply any preprocessing step to improve the quality of the images and we did not change their original size.

## Conclusions

In this paper, we presented a novel approach for the grading of invasive breast carcinoma by applying histological image classification on the Grassmann manifold. More specifically, we showed that breast cancer histological images can be considered as a set of multidimensional spatially-evolving signals, which can be efficiently modeled through a higher-order linear dynamical systems analysis, and then we proposed the VLAD encoding of each image on the Grassmannian space. The key advantage of the proposed method over existing methods is the fact that it exploits both image dynamics and appearance information, while at the same time it avoids the detection of the histologic primitives, such as nuclei, which is usually a challenging task due to the complex appearance of the tissue. Experimental results using two datasets with different characteristics showed the superiority of the proposed method against a number of state of the art approaches (14 different state of the art methods) based on either handcrafted features, such as textural, graph or morphological, or deep learning. In the future, we aim to explore its applicability to other histological image classification problems, exploiting the fact that the proposed method is based on the direct modeling of data rather than on any previous domain knowledge.
